# Monitoring of Physiological Parameters to Predict Exacerbations of Chronic Obstructive Pulmonary Disease (COPD): A Systematic Review

**DOI:** 10.3390/jcm5120108

**Published:** 2016-11-25

**Authors:** Ahmed M. Al Rajeh, John R. Hurst

**Affiliations:** UCL Respiratory, Royal Free Campus, University College London, London NW3 2PF, UK; j.hurst@ucl.ac.uk

**Keywords:** COPD, exacerbation, physiological signs, vital signs, lung function, home monitoring, telehealth

## Abstract

Introduction: The value of monitoring physiological parameters to predict chronic obstructive pulmonary disease (COPD) exacerbations is controversial. A few studies have suggested benefit from domiciliary monitoring of vital signs, and/or lung function but there is no existing systematic review. Objectives: To conduct a systematic review of the effectiveness of monitoring physiological parameters to predict COPD exacerbation. Methods: An electronic systematic search compliant with Preferred Reporting Items for Systematic Reviews and Meta-Analyses (PRISMA) guidelines was conducted. The search was updated to April 6, 2016. Five databases were examined: Medical Literature Analysis and Retrieval System Online, or MEDLARS Online (Medline), Excerpta Medica dataBASE (Embase), Allied and Complementary Medicine Database (AMED), Cumulative Index of Nursing and Allied Health Literature (CINAHL) and the Cochrane clinical trials database. Results: Sixteen articles met the pre-specified inclusion criteria. Fifteen of these articules reported positive results in predicting COPD exacerbation via monitoring of physiological parameters. Nine studies showed a reduction in peripheral oxygen saturation (SpO_2_%) prior to exacerbation onset. Three studies for peak flow, and two studies for respiratory rate reported a significant variation prior to or at exacerbation onset. A particular challenge is accounting for baseline heterogeneity in parameters between patients. Conclusion: There is currently insufficient information on how physiological parameters vary prior to exacerbation to support routine domiciliary monitoring for the prediction of exacerbations in COPD. However, the method remains promising.

## 1. Introduction

Chronic obstructive pulmonary disease (COPD) is a serious health matter, which significantly impacts the individual’s quality of life. According to the World Health Organisation, in 2004, 65 million people were diagnosed with COPD globally [1]. In 2012, three million people died because of COPD [2], and thus COPD is anticipated to be the third leading cause of death by 2020 if no action is taken [3]. COPD, even when optimally managed, is associated with periodic deteriorations in respiratory health called exacerbations. Exacerbations are defined in the Global Initiative for Chronic Obstructive Lung Disease (GOLD) document “*as an acute event characterised by a worsening of the patient’s respiratory symptoms that is beyond normal day-to-day variations and leads to a change in medication*” [4]. Exacerbations can lead to decline in the patient’s overall function, causing hospitalisation, and/or death. Therefore, health care facilities, societies, and individuals have a common interest in better understanding how to prevent and manage exacerbations, reduce disease progression, and support patient self-management. To achieve this, early detection of exacerbations and prompt access to therapy and health services are needed. Detecting COPD exacerbation earlier will allow prompt initiation of treatment [4]; therefore facilitating faster recovery and outcomes. This may result in a reduced number of hospital admissions, and as well as a reduction in healthcare consumption.

It is recognised that whilst defined by changes in symptoms, exacerbations are also associated with alterations in physiological variables. In 2010, Hurst et al. [5] examined the ability of domiciliary pulse oximetry and peak flow to distinguish exacerbations from day to day fluctuations. They reported that changes in heart rate, peripheral oxygen saturation (SpO_2_%), and peak flow were significantly different just before and during an exacerbation. Rapid advancement in technology has offered numerous solutions targeting the management of chronic diseases (collectively known as tele-health). Tele-health is a form of distance communication between the patient and the healthcare provider for monitoring, communicating, managing or facilitating intervention [6]. Tele-health may monitor symptoms, and/or physiology parameters. Tele-health has shown some success in chronic disease management. The PROMETE study conducted in 2014 in Spain by Segrelles et al. reported a reduction in acute noninvasive ventilation (NIV) usage (*p* < 0.0001), emergency department (ER) visits (*p* = 0.001), admissions (*p* = 0.015) and bed days (*p* = 0.018) [7]. More recent studies in COPD have not been positive [8], perhaps reflecting the heterogeneity of COPD.

The objective of this systematic review was to summarise and report the value of domiciliary physiological monitoring in predicting exacerbations in patients with COPD.

## 2. Methods

### 2.1. Search Strategy

This systematic review (PROSPERO registration CRD42016046643) is compliant with the Preferred Reporting Items for Systematic Reviews and Meta-Analyses (PRISMA) standards [9]. The search was completed up to April 6, 2016. The search was performed in Medical Literature Analysis and Retrieval System Online, or MEDLARS Online (Medline), Excerpta Medica dataBASE (Embase), Allied and Complementary Medicine Database (AMED), Cumulative Index of Nursing and Allied Health Literature (CINAHL), and the **Cochrane** clinical trials database. The search terms used are detailed in the Appendix, Table A1 and Table A2. In addition to the electronic database search, the reference list of eligible articles was also screened.

### 2.2. Inclusion Criteria

The studies included in this review met the following criteria: (1) Stable COPD; (2) Domiciliary monitoring; (3) Monitoring any physiological variables; (4) Reporting statistical analysis of the measured physiological variables; (5) Prediction of exacerbations via physiological variables.

### 2.3. Exclusion Criteria

We excluded the following: (1) Books; (2) Systematic reviews; (3) Non-English manuscripts; (4) Conference abstracts with no full-text; (5) Non-full text articles.

The main outcome of interest was variation in physiological parameters before and during COPD exacerbations, and the ability of measuring changes in physiological variables to provide early detection of COPD exacerbations.

### 2.4. Data Collection

Screening of the titles and abstracts was performed by the first author to eliminate all non-relevant studies. Titles and abstracts potentially relevant were read in full-text to evaluate if they were eligible or not. In addition to screening and evaluating for eligibility, the reference list of the eligible articles was screened. The second author confirmed the eligibility. Disagreement on five studies between authors was resolved after discussion.

### 2.5. Quality Assessment

The quality assessment was performed by each author individually based on two different modified scales, the Cochrane tool [10] and Newcastle-Ottawa scale [11]. The Cochrane quality assessment tool consists of seven questions to evaluate randomised studies included in this review. The Newcastle-Ottawa scale consists of seven questions used to assess cohort and non-randomised studies included in this review. The assessment was performed by each author individually and any disagreement was solved by discussion.

### 2.6. Synthesis of Results

The primary purpose of this systematic review was to assess the feasibility of predicting COPD exacerbations by domiciliary monitoring of physiological parameters. Because of significant methodological heterogeneity between included studies, meta-analysis was not conducted. However, a narrative synthesis of the results of the studies was performed and full details of the included studies are reported in Table 1 and Table 2.

## 3. Results

**Figure 1:**
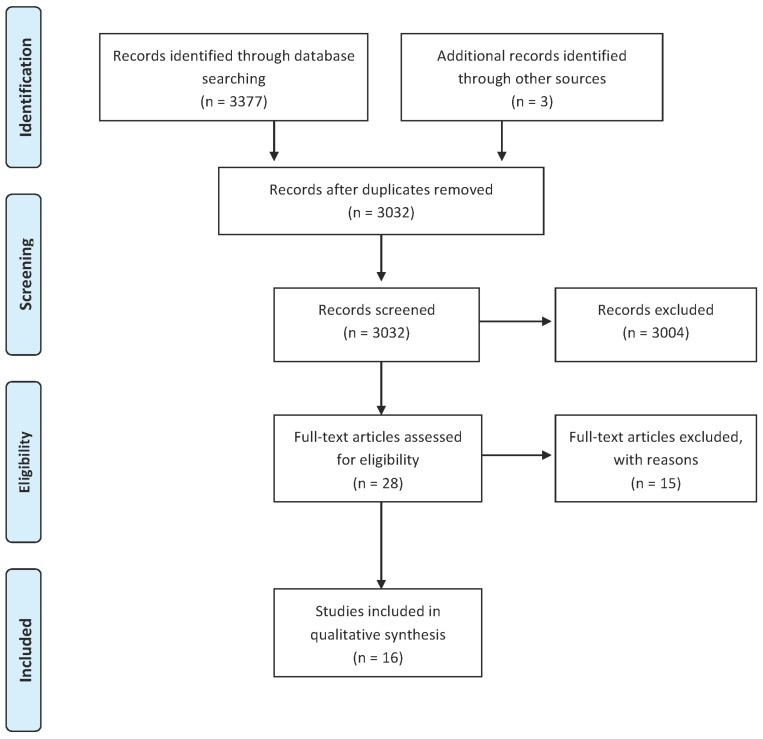
Preferred Reporting Items for Systematic Reviews and Meta-Analyses (PRISMA) Flow Diagram.

The systematic review search generated 3377 articles, 345 were excluded due to duplication. After screening the titles and abstracts, 28 articles out of 3032 were potentially relevant to the inclusion criteria. After that, full-text screening of the 28 articles was conducted to assess eligibility, which resulted in 13 relevant articles. The reference list of the relevant articles was also examined which resulted in identification of three further articles giving 16 in total (Figure 1).

Of the 16 articles that met the pre-specified inclusion criteria, all the studies were conducted prospectively, and in seven different countries: one each in Australia, Denmark, France, Italy, Netherlands, four in Spain, and eight in the United Kingdom. Most of the articles were published in 2015 (5/16), with three in 2012, two each in 2009 and 2013, and one each was published in 2000, 2010, 2014 and 2016. The sample size and duration of the studies varied from three months to fifteen months except for one study, which was run for 30 months. The sample size varied from 16 to 183 participants (eight studies <50 patients, five studies ≥50 patients, and three studies >100 patients). Fifteen studies were on COPD patients only (at different disease stages), and one was on heart failure and chronic lung disease patients [16]. Full details of the included studies are reported in Table 1 and Table 2.

### Quality Assessment

Among the 16 identified articles, four studies were randomised clinical trials and 12 were cohort studies. The four studies evaluated using the modified Cochrane risk of bias tool [10] were ranked as being at high risk of bias. The 12 studies evaluated by the modified Newcastle-Ottawa scale [11] were all ranked as moderate quality except for one, which was ranked as low quality.

## 4. Monitoring Vital Signs to Predict Exacerbation

### 4.1. Heart Rate and Oxygen Saturation

Most of the included studies 14/16 monitored the participant’s vital signs and assessed the capability of vital signs to predict COPD exacerbation. Although heart rate (HR) and oxygen saturation (SpO_2_%) were monitored in 10/16 studies [5,7,13,15,18,19,20,21,23,25], 7/10 studies did not report any statistical analysis for the HR and SpO_2_% variation. However, they concluded with the possibility that heart rate and/or SpO_2_% may be useful in detecting deterioration. Four studies (three at moderate quality, and one at high risk of bias) reported a significant variation (*p* ≤ 0.05) in HR and/or SpO_2_% prior to the onset of COPD exacerbation [5,18,23,25]. In Hurst et al. [5], the magnitude of the fall in SpO_2_% two days into the exacerbation was −1.24 standard deviation (SD) and the rise in HR was +3.09 SD above the patient’s baseline. Martin-Lesende et al. [18] reported the difference between the mean values monitored over the whole study period, which were for SpO_2_% 93.1% (2.2 SD), and for HR 77.8 min^−1^ (14.6 SD); Moreover, the mean values monitored over the five days prior to cause-specific admission were for SpO_2_% 91.0% (4.6 SD) and for HR 84.2 min^−1^ (17.1 SD), *p* = 0.003 for both. There was therefore a typical rise in HR of 7 min^−1^ and fall in SpO_2_% of 2%. Burton et al. [23] reported that the magnitude of SpO_2_% fall and HR rise was approximately 1 SD (SpO_2_% fall from 93.6% to 92.4%, and HR increased from 87.4 min^−1^ to 93.7 min^−1^).

### 4.2. Respiratory Rate

The works of Yanez and Borel, which were ranked as moderate quality [17,24], evaluated variations in respiratory rate prior to an exacerbation. In both, the change was statistically significant (*p* ≤ 0.05). Importantly Yanez et al. reported an increase in the mean respiratory rate one to five days prior to hospitalisation due to an acute exacerbation. At 48 h, the mean respiratory rate increased by 2.3 min^−1^ (15% from baseline) with 72% sensitivity and 77% specificity (area under the curve (AUC) = 0.76, *p* < 0.05) for detecting exacerbation, whilst the rise noted 24 h prior to hospitalisation at 4.4 min^−1^ (30% from baseline) had a 66% sensitivity and 93% specificity (AUC = 0.79, *p* < 0.05) for exacerbation detection. At five days before hospitalisation, the mean respiratory rate rose from 15.2 ± 4.3 min^−1^ to 19.1 ± 5.9 min^−1^ (*p* < 0.05) suggesting a longer window for preventing hospitalisation. However, in contrast, Martin Lesende [18] did not see significant change in the respiratory rate five days before hospitalisation. Mohktar [21] included respiratory rate with daily monitored variables, but no analysis was reported.

### 4.3. Blood Pressure and Temperature

Four studies of 16 (two at high risk of bias and two at moderate quality) included blood pressure monitoring [7,15,18,21], but there was no evidence indicating changes in blood pressure was as a variable with high predictive capacity for exacerbation (*p*-value not significant). Likewise, body-temperature was monitored in 4 out of of 16 studies. Martin-Lesende [18] compared the mean temperature in the overall follow-up period, 35.9 °C (0.4SD), to the mean of five days, 35.5 °C (1 SD), prior to cause-specific admission. Changes in body temperature resulted in 27.8% of alerts (only 5.6% of alerts were due to an increased temperature over 37 °C). Hamad [25] reported increased body-temperature in 9 out of 98 exacerbations.

Five studies (two at high risk of bias and three at moderate quality) out of 16 [13,15,19,20,22] did not provide sufficient statistical analysis of changes in vital signs despite reporting these variables. For example, Pedone [19] evaluated the capability of a tele-monitoring system for lower hospitalisation rates, and to identify COPD exacerbation onset. The researchers did not report whether the result was statistically significant but noted a 33% reduction in the risk of hospitalisation. Pedone also noted a fall in SpO_2_% in three days preceding the onset of an exacerbation, which therefore led to prediction of COPD exacerbation. Furthermore, Jensen [15] tried to develop an algorithm to enhance the prediction of COPD exacerbation. The four variables heart rate, systolic blood pressure, diastolic blood pressure, and oxygen saturation were monitored and classified into 273 features. Jensen reported that their system was able to distinguish ten COPD exacerbations with 70% sensitivity, 95% specificity, and 0.73 AUC.

Considered together, SpO_2_% was the most studied variable before an exacerbation episode, and the variable which has been reported to have the highest predictive capacity although the magnitude of change is typically small (1%–2%).

## 5. Monitoring Lung Function to Predict Exacerbations

Lung function, particularly spirometry, is a valuable test for diagnosing COPD and evaluating disease progression. A few studies assessed the usefulness of lung function variables in predicting acute exacerbation. Eight studies (two at high risk of bias, one as low quality, and two at moderate quality) of 16 [5,7,12,13,14,16,21,23] monitored either the peak expiratory flow rate (PEFR), or the forced expiratory volume in one second (FEV_1_), or both. Three studies [12,13,23] measured FEV_1_ and PEFR at different frequencies (per day/per week). Seemungal et al. [12] reported data from 101 COPD patients on PEFR, FEV_1_ and vital capacity (FVC) on the day of exacerbation onset, and showed significant changes (*p* < 0.001). The analysis of 504 COPD exacerbations revealed a fall in the median PEFR of 8.6 (interquartile range (IQR) 0 to 22.9) L/min, FVC of 76.0 (IQR −40.4 to 216.4) mL, and FEV_1_ of 24.0 (IQR −16.1 to 84.3) mL. Burton et al. [23] reported a strong correlation between FEV_1_ and PEFR and a 0.1 L reduction in FEV_1_ was associated with a change in the symptom score.

Sund et al. at low quality and Mohktar et al. at moderate quality [14,21] focused only on FEV_1_. Sund [14] detected 55/75 exacerbations using monitoring, and 6/55 exacerbations were detected only via FEV_1_ (defined as a 10% fall in FEV_1_ for ≥2 consecutive days). Three studies [5,7,16] examined predicting COPD exacerbations with daily monitoring of PEFR. Segrelles [7] did not report detailed PEFR data, but reported that PEFR and SpO_2_% were the most predictive variables. Hurst [5] reported a statistically significant variation in PEFR before and during an acute exacerbation with a maximal −2.97 SD fall in PEFR four days into the exacerbation. However, Berge [16] reported a significant decrease in the mean of PEFR during an exacerbation episode, which was back to baseline in two weeks.

## 6. Monitoring Respiratory Sounds to Predict Exacerbations

In 2015 Fernandez-Granero at moderate quality [22] reported a study demonstrating that 25 out of 33 COPD exacerbations could be detected via monitoring patient’s respiratory sounds at home. Each participant was asked to record his/her respiratory sounds daily by placing a microphone on the suprasternal notch. Exacerbation episodes were detected 5 ± 1.9 days prior to the exacerbation onset with a sensitivity of 73.76% and 97.67% specificity.

## 7. Methodological Considerations

### 7.1. Alarm limits

A challenge in COPD is the variation between patients and how to set alarm limits for an individual patient. Of the 16 articles included in this review, only eight studies (three at high risk of bias, one at low quality and two at moderate quality) [5,13,14,18,19,20,21,25] mentioned that they had customised the alarm limits for each individual. Methods used were reported in six out of the eight studies. Cooper [13] monitored the participants for two weeks to identify the normal range for each and personalise the alert limits. Sund [14] set a baseline for each participant by taking the median and the mean after monitoring symptoms and FEV_1_ for 14 days (exacerbation-free). In the Hurst study [5], heart rate, oxygen saturation, and peak expiratory flow rate assessed for 30 days (symptom-free). These established a baseline of the selected variables with ±SD. Pedone [19] customised the limits based on the participant’s “clinical situation”. Harding [20] personalised each participant’s limits by applying a probability density function after monitoring the participant for six weeks, or having 40 sets of recorded daily data. Mokhtar [21] personalised the limits range in a different way; they took the median (50th percentile), lower (25th percentile), and upper (75th percentile). They then adjusted the lower limits to be 25th percentile minus 1.5 times the interquartile, and the upper limits to be 75th percentile plus the 1.5 times the interquartile. There are no studies comparing different methods of personalising alarm limits.

### 7.2. Monitoring Characteristics

The approach pursued by the 16 studies in monitoring physiological signs were heterogeneous with regard to the type of equipment or instrument used to monitor and assess the participant’s data. In some studies, a mobile/tablet app was used to communicate with the participant [19,20], and transfer data. Some studies set up a monitoring station for each individual with different devices [7,13,14,15,17,18,19,21,22,23,24,25], where the data were transmitted automatically through an Internet modem. If a red flag was raised or threshold breached, a notification alert was sent to the system operator in real time. In two other studies, another form of monitoring was used. A diary card for symptoms and vitals were provided to participants, and a visit was arranged to collect the data [5,12,16].

### 7.3. Intermittent vs. Continuous Monitoring

In the reviewed articles, 16 studies monitored the participants’ physiological parameters and symptoms intermittently. The frequency of monitoring/recording was varied, some once daily or multiple times daily. However, in four studies [7,13,14,23], participant’s data were monitored less than daily (different frequencies per week). In addition to that, sometimes measurements taken were restricted to morning, however, in Harding et al. [20], the stipulated time for measurements recording was based on the patient’s preference.

## 8. Discussion

We have conducted the first systematic review examining the utility of monitoring physiological variables to predict exacerbations of COPD. In general, and as discussed below, the studies are small and heterogeneous using different variables and different protocols. The need for better healthcare solutions in people diagnosed with chronic diseases is real. COPD imposes burdens on individuals and health care organisations. Whilst the methods hold promise, further adequately powered studies are required to properly define the utility of physiological monitoring to predict exacerbations.

In this systematic review, sixteen articles met the inclusion criteria, which were compliant with PRISMA. Five studies out of 16 [13,15,19,20,22] did not provide sufficient statistical data to draw conclusions consistent with the results of other studies, despite reporting changes in physiological variables (no *p*-value). The methodological quality of the studies was variable but generally low with 12 cohort studies ranked as moderate or low quality, and four trials ranked as having a high risk of bias.

We have described those studies that showed positive results in predicting/detecting an exacerbation episode via monitoring of physiological parameters. Although this approach appears to be promising, further well-designed clinical trials are required to investigate the true magnitude and time-course pre, during, and post an exacerbation episode of changes in physiological parameters. Understanding the extent of the magnitude of change for each variable is critical in using this knowledge for early exacerbation detection. In three studies [5,18,23] the magnitude of the change in heart rate and SpO_2_% reported was an increase of around 5 min^−1^ for heart rate and a fall by 1%–2% for SpO_2_%. Two studies [17,24] reported an increase in the respiratory rate before the onset of COPD exacerbation/hospitalisation. These findings all support the hypothesis that monitoring of vital signs can detect respiratory deterioration. However, the question arises as to whether these variables can be reliable enough. Moreover, to answer that question we need to better understand the relationship between physiological signs and symptoms. This has been confirmed in some of the above mentioned studies [5,12,14]. Hurst combined peak expiratory flow (PEF) with a symptom score to provide optimal exacerbation detection [5].

Having demonstrated that monitoring physiological variables has the theoretical potential to detect COPD exacerbations, the second step is implementation of this in a clinical environment—Tele-monitoring. To enable healthcare providers and patients to feel secure managing COPD and detecting acute exacerbations with no anticipated harm, an intelligent interface to provide live communication is essential. In the above mentioned studies, various designs were employed. However, the optimal technique/algorithm still requires more investigation. Despite the fact that tele-health offers the possibility for the clinician and the patient to be connected and monitored in a ‘virtual clinic’, the accuracy and specificity of this discipline are still uncertain. Developing an algorithm to detect an exacerbation is important because that would facilitate the services provided via tele-health. A particular challenge is around alarm thresholds. To increase the value of tele-health in self-management, a customised threshold for each patient is essential as this will help to decrease false alarms, and differentiate between true deterioration and day-to-day variation. Six studies had addressed this issue by specifying the alarm settings for each individual [5,13,14,19,20,21], but using different methods and the optimal way to set individual patient alarms remains an open question.

Even though most of the reviewed studies exhibited some significant positive results in the efficacy of physiological parameters in predicting/detecting COPD exacerbation, there are insufficient data to draw a secure conclusion in this review. This is due to the diversity of the designs, methods, and sample size of studies. The demand for technology to meet the needs of the COPD patient and society are increasing. Further clinical trials are needed to achieve that.

### Strength and Limitations

In this systematic review, a number of limitations can be considered. First, non-English studies (abstract and full text) were excluded. Second, only one author performed the screening of titles and abstracts, which may have increased the risk that studies were excluded inappropriately. Thirdly, the definitions of exacerbation vary across the studies, which can make comparison between studies challenging. The major strength of this study is that, to our knowledge, there is no pre-existing review conducted regarding the usefulness of monitoring physiological signs to predict COPD exacerbation.

## 9. Conclusions

Monitoring of physiological parameters may be useful in assisting earlier detection of COPD exacerbations but further, robust studies are required to confirm this. A particular challenge is how to set alarm limits for individual patients given the heterogeneity inherent in COPD and COPD exacerbations.

## Figures and Tables

**Table 1:** Detailed description of the 16 included studies.

Author	Subjects and COPD Severity	Country	Measures	Quality	Detailed Description	Results
**Seemungal et al., 2000 [12]**	*N* = 101 severe COPD	United Kingdom	PEFR FEV_1_ Symptoms	**Moderate quality**	**Period**: 2.5 years. PEFR and symptoms measured daily, post morning medication. In a subgroup of 34, FEV_1_ was measured	Analysis of 504 exacerbations: Lung function changed significantly on the day of onset (*p* < 0.001). A decrease in the median of: PEFR by 8.6 L/m FEV_1_: 24.0 mL FVC: 76.0 mL
**Cooper et al., 2009 [13]**	*N* = 19 mild−severe COPD	United Kingdom	HR SpO_2_% PEFR FEV_1_ Symptoms	**High risk of bias**	**Period**: 4 months. Participants measured their vital signs and recorded their symptoms twice a week in the morning	Analysis of four exacerbations: Concluded that SpO_2_% was the variable most closely associated with exacerbation but no statistical significance reported
**Sund et al., 2009 [14]**	*N* = 18 severe COPD	United Kingdom	FEV_1_ Symptoms	**Low quality**	**Period**: 6 months. Daily electronic diary and performed three spirometry manoeuvres daily in the evening	Analysis of 75 exacerbations: 55 exacerbations were detected via tele-health (symptoms) and 6/55 exacerbations were detected via FEV_1_ alone (*p* = not significant). Exacerbation detected via FEV_1_ was defined as a 10% fall in FEV_1_ for ≥2 consecutive days.
**Hurst et al., 2010 [5]**	*N* = 31 severe COPD	United Kingdom	HR SpO_2_% PEFR Symptoms	**Moderate quality**	**Period**: 9 months. Daily paper diary cards	Analysis of 13 exacerbations: Variation was noted prior and during the onset of exacerbation in PEFR, HR, and SpO_2_%. Maximal change in SpO_2_% and HR occurred two days into exacerbation: SpO_2_% had fallen by −1.24 SD, HR increased by +3.09 SD. Maximal change in PEFR occurred four days into exacerbation: −2.97SD Composite Score to detect exacerbation: AUC = 0.832, *p* < 0.05.
**Jensen et al. in 2012 [15]**	*N* = 57 moderate−severe COPD	Denmark	HR SpO_2_% BP	**Moderate quality**	**Period**: 4 months. Daily diary cards	Analysis of 9 exacerbations: Their algorithm classified variables into 273 features and was able to detect seven exacerbations via vital signs with 70% sensitivity, 95% specificity, AUC = 0.73.
**Berge et al., 2012 [16]**	*N* = 137 severe COPD	Netherlands	Salbutamol use PEFR Symptoms	**Moderate quality**	**Period**: 15 months. Daily diary cards	Analysis of 101 exacerbations: Significant decrease in PEFR 15 L/min at exacerbation compared to baseline.
**Yanez et al. in 2012 [17]**	*N* = 89 severe COPD (On O_2_ therapy)	Spain	Respiratory Rate (RR)	**Moderate quality**	**Period**: 3 months. Daily monitoring of respiratory rate, using a sensor inserted into the domiciliary oxygen supply system	Analysis of 10 exacerbations: Increase in the mean respiratory rate in 21/30 exacerbations, 1–5 days prior to hospitalisation Mean of respiratory rate raised: Five days: 15.2 ± 4.3 min^−1^ to 19.1 ± 5.9 min^−1^ Two days: 2.3 min^−1^ (15% from baseline) One day: 4.4 min^−1^ (30% from baseline) All *p*-value < 0.05
**Martin Lesende et al. 2013 [18]**	*N* = 58 Heart failure (27.6%) + O_2_ therapy (57.1%) + moderate−very severe COPD and asthma 25.9%	Spain	HR SpO_2_% BP RR Weight Temperature Symptoms	**High risk of bias**	**Period**: 12 months. Daily monitoring	In the five days preceding hospital admission: Mean SpO_2_% fell from 93.1% to 91.0% (4.6 SD), and mean HR increased from 77.8 to 84.2 min^−1^ (17.1 SD) *p* = 0.003 for both. No significant change for respiratory rate, body temperature and blood pressure.
**Pedone et al. 2013 [19]**	*N* = 99 moderate−severe COPD	Italy	HR SpO_2_% TemperaturePhysical activity	**High risk of bias**	**Period**: 9 months. Automatic recording of vital signs, a mean of four times per day.	Analysis of 13 exacerbations: SpO_2_% fell three days before an exacerbation, which permitted timely intervention, and was associated with a 33% reduction in hospitalisation rate (*p* = not shown, data displayed in a Figure only).
**Segrelles et al., 2014 [7]**	*N* = 60 severe COPD (On O_2_ therapy)	Spain	HR SpO_2_% BP PEFR	**High risk of bias**	**Period**: 7 months. Participants monitored their vital signs every morning, but PEFR was three times/week.	Analysis of 50 red flags: confirmed red flag defined as moderate, severe or very severe exacerbation. Tele-health was associated with significant reduction in acute NIV usage (*p* < 0.0001), ER visits (*p* = 0.001), admissions (*p* = 0.015) and bed days (*p* = 0.018). Reported that SpO_2_% and PEFR were the most predictive parameters (but data not reported).
**Harding et al., 2015 [20]**	*N* = 18 moderate−very severe COPD	United Kingdom	HR SpO_2_% Symptoms	**Moderate quality**	**Period**: 6 months. Each participant asked to fill a daily symptom diary card.	Analysis of 37 exacerbations: 15/37 exacerbations were identified in three days prior to medication self-initiation.Alerts related to events: 47 symptom alerts (16 patients)80 HR alerts (18 patients), and 62 SpO_2_% alerts (17 patients). *p* = not shown.
**Mohktar et al., 2015** [21]	*N* = 21 moderate−very severe COPD	Australia	HR SpO_2_% BP RR Weight Temperature FEV_1_ Symptoms	**Moderate quality**	**Period**: 11 months. Participants daily monitored their vital signs and symptoms	Analysis of 90 exacerbations: The designed algorithm identified 55/90 true exacerbations (71.8% sensitivity 80.4% specificity). FEV_1_ value (k = 0.21), mean of distribution of SpO_2_% (k = 0.27) and the weight (k = 0.21) were the most predictive variables (*p* = not shown).
**Fernandez-Granero et al., 2015 [22]**	*N* = 16 severe−moderate COPD	Spain	Respiratory sound	**Moderate quality**	**Period**: 6 months. Daily recorded respiratory sounds using a microphone over the super-sternal notch	Analysis of 33 exacerbations: 25 out of 33 exacerbations were detected 5 ± 1.9 days prior to the onset of exacerbation by changes in sounds (*p* = not shown).
**Burton et al., 2015** [23]	*N* = 33 mild−very severe COPD	United Kingdom	HR SpO_2_% FEV_1_ PEFR Symptoms	**Moderate quality**	**Period**: >200 days. Each participant asked to fill a symptom questionnaire, and measure heart rate, and SpO_2_% daily. FEV_1_ and PEFR monitored weekly.	Analysis of 172 exacerbations: Increase in HR (87 min^−1^–94 min^−1^) at the onset of exacerbation and mean SpO_2_% fell (93.6% to 92.4%) around the onset of exacerbation. Exacerbation associated with a reduction of 0.1 L in FEV_1_.
**Borel et al., 2015 [24]**	*N* = 44 severe COPD (On NIV and O_2_ therapy)	France	RR %Triggering NIV usage Questionnaire	**Moderate quality**	**Period**: 6 months. Daily monitoring via the ventilator and daily EXACT-Pro questionnaire.	Analysis of 21 exacerbations: 21 exacerbations detected, and the risk of exacerbation was high if high value noted on ≥ two days out of five for RR P = 0.01, and %Triggered Breaths *p* = 0.037, but not total NIV usage *p* = 0.097).
**Hamad et al., 2016 [25]**	*N* = 183 COPD *	United Kingdom	HR SpO_2_% Temperature Physical activity Symptoms	**Moderate quality**	**Period**: 4 months. Daily monitoring.	Analysis of 98 exacerbations: 80/98 showed changes on one or more tele-health parameters prior to hospitalisation/exacerbation onset. 30 exacerbations resulted in hospitalisation and 7/30 had significant SpO_2_% reduction (significant defined for each patient individually, *p* = 0.049) 12/98 exacerbations had a significant SpO_2_% fall (*p* < 0.05).

* Disease severity not reported. COPD: chronic obstructive pulmonary disease; FVC: forced vital capacity; PEFR: peak expiratory flow rate; FEV_1_ forced expiratory volume in one second; HR: heart rate; SpO_2_%: peripheral capillary oxygen saturation; BP: blood pressure; RR: respiratory rate; NIV: noninvasive ventilation; EXACT: exacerbations of chronic pulmonary disease tool; Pro: Patient-reported outcome; SD: standard deviation; AUC: area under the curve.

**Table 2:** Detailed description of the 16 included studies.

Author	Definition of Exacerbation
Seemungal et al., 2000 [12]	Anthonisen criteria.
Cooper et al., 2009 [13]	Not explained.
Sund et al., 2009 [14]	Increase of two symptoms and/or ≥10% reduction of FEV_1_ for ≥2 consecutive days; or the use of antibiotics and/or prednisolone.
Hurst et al., 2010 [5]	≥2 of new or worsening symptoms (one should be increased breathlessness, sputum volume of sputum purulence) for ≥2 days.
Jensen et al. in 2012 [15]	Admission to hospital, or started antibiotics or steroids with specific symptoms.
Berge et al., 2012 [16]	Not explained.
Yanez et al., 2012 [17]	Clinical diagnosis by an emergency room clinician.
Martin Lesende et al., 2013 [18]	Not explained.
Pedone et al., 2013 [19]	Change in symptoms that lead to a change in medication.
Segrelles et al., 2014 [7]	GOLD definition.
Harding et al., 2015 [20]	Initiation of antibiotics or steroids or both.
Mohktar et al., 2015 [21]	GOLD definition.
Fernandez-Granero et al., 2015 [22]	Use of medication for exacerbation, and/or unplanned emergency room visits and/or hospital admissions.
Burton et al., 2015 [23]	Anthonisen criteria or started antibiotics.
Borel et al., 2015 [24]	If abnormal values of respiratory rate and % triggered breaths were reported for two days or more, or abnormal values of NIV daily usage were reported for three days or more out of five. Abnormal values were defined as “value of a parameter was >75th or <25th percentile, the day was recorded as abnormal value’ (‘high value’ > 75th, ‘low value’ < 25th).
Hamad et al., 2016 [25]	Admission to hospital, or started antibiotics or/and steroids.

## Appendix A

**Table A1:** Medline Search Strategy.

1	lung diseases, obstructive/ or exp. bronchitis/ or exp. pulmonary disease, chronic obstructive/	84,036	Advanced	Display Results More
2	emphysema$.mp.	31,994	Advanced	Display Results More
3	bronchiti$.mp.	30,780	Advanced	Display Results More
4	(obstruct$ adj3 (pulmonary or lung$ or airway$ or airflow$ or bronch$ or respirat$)).mp.	96,121	Advanced	Display Results More
5	(copd or coad or cobd or aecb).mp. [mp = title, abstract, original title, name of substance word, subject heading word, keyword heading word, protocol supplementary concept word, rare disease supplementary concept word, unique identifier]	33,479	Advanced	Display Results More
6	1 or 2 or 3 or 4 or 5	153,864	Advanced	Display Results More
7	telemedicine/ or telerehabilitation/	14,118	Advanced	Display Results More
8	(telemonitor* or tele-monitor* or tele-health* or telehealth* or telemedicine or tele-medicine or tele-rehabilitat* or telerehabilitat*).mp. [mp = title, abstract, original title, name of substance word, subject heading word, keyword heading word, protocol supplementary concept word, rare disease supplementary concept word, unique identifier]	18,772	Advanced	Display Results More
9	(e-health or ehealth or m-health or mhealth or mobile health).mp. [mp = title, abstract, original title, name of substance word, subject heading word, keyword heading word, protocol supplementary concept word, rare disease supplementary concept word, unique identifier]	8219	Advanced	Display Results More
10	exp. Telemetry/	10,614	Advanced	Display Results More
11	(telemetr* or tele-metr*).mp. [mp = title, abstract, original title, name of substance word, subject heading word, keyword heading word, protocol supplementary concept word, rare disease supplementary concept word, unique identifier]	12,888	Advanced	Display Results More
12	Monitoring, Ambulatory/	6635	Advanced	Display Results More
13	(monitoring adj4 (ambulatory or home$)).mp. [mp = title, abstract, original title, name of substance word, subject heading word, keyword heading word, protocol supplementary concept word, rare disease supplementary concept word, unique identifier]	23,156	Advanced	Display Results More
14	Domiciliary.mp.	2364	Advanced	Display Results More
15	software/ or mobile applications/ or user-computer interface/	114,192	Advanced	Display Results More
16	(software* or app? or iphone or ipad or android or smartphone* or smart-phone*).mp. [mp = title, abstract, original title, name of substance word, subject heading word, keyword heading word, protocol supplementary concept word, rare disease supplementary concept word, unique identifier]	205,344	Advanced	Display Results More
17	or/7–16	284,600	Advanced	Display Results More
18	(exacerbat* or deteriorat*).mp. [mp = title, abstract, original title, name of substance word, subject heading word, keyword heading word, protocol supplementary concept word, rare disease supplementary concept word, unique identifier]	176,862	Advanced	Display Results More
19	heart rate/	149,127	Advanced	Display Results More
20	Pulse/	16,765	Advanced	Display Results More
21	((heart* or pulse* or cardiac) adj3 rate*).mp.	229,964	Advanced	Display Results More
22	respiratory rate/ or Respiration/	75,932	Advanced	Display Results More
23	((respirat* or breath*) adj3 rate*).mp. [mp = title, abstract, original title, name of substance word, subject heading word, keyword heading word, protocol supplementary concept word, rare disease supplementary concept word, unique identifier]	24,774	Advanced	Display Results More
24	exp. Oximetry/	13,116	Advanced	Display Results More
25	oximetr*.mp.	15,161	Advanced	Display Results More
26	Oxygen/	150,124	Advanced	Display Results More
27	SPO_2_.mp.	3207	Advanced	Display Results More
28	oxygen.mp.	519,842	Advanced	Display Results More
29	(physiological adj4 (variable* or measure*)).mp. [mp = title, abstract, original title, name of substance word, subject heading word, keyword heading word, protocol supplementary concept word, rare disease supplementary concept word, unique identifier]	10,547	Advanced	Display Results More
30	early diagnosis/	19,913	Advanced	Display Results More
31	(early adj4 (detect* or diagnos*)).mp. [mp = title, abstract, original title, name of substance word, subject heading word, keyword heading word, protocol supplementary concept word, rare disease supplementary concept word, unique identifier]	176,122	Advanced	Display Results More
32	predict*.mp. [mp = title, abstract, original title, name of substance word, subject heading word, keyword heading word, protocol supplementary concept word, rare disease supplementary concept word, unique identifier]	1,238,846	Advanced	Display Results More
33	or/18–32	2,291,354	Advanced	Display Results More
34	6 and 17 and 33	795	Advanced	

**Table A2:** Database Search Strategy.

Database	Subject Heading	Keyword
Medline	lung diseases, obstructive/ or exp. bronchitis/ or exp. pulmonary disease, chronic obstructive/ telemedicine/ or telerehabilitation/ exp. Telemetry/ Monitoring, Ambulatory/ software/ or mobile applications/ or user-computer interface/ heart rate/ Pulse/ respiratory rate/ or Respiration/ exp. Oximetry/ Oxygen/ early diagnosis/	emphysema$. bronchiti$. (obstruct$ adj3 (pulmonary or lung$ or airway$ or airflow$ or bronch$ or respirat$)). (copd or coad or cobd or aecb) (telemonitor* or tele-monitor* or tele-health* or telehealth* or telemedicine or tele-medicine or tele-rehabilitat* or telerehabilitat*) (telemetr* or tele-metr*) (monitoring adj4 (ambulatory or home$)). Domiciliary. (software* or app? or iphone or ipad or android or smartphone* or smart-phone*). (exacerbat* or deteriorat*). ((heart* or pulse* or cardiac) adj3 rate*). ((respirat* or breath*) adj3 rate*). oximetr*. SPO_2_. oxygen. (physiological adj4 (variable* or measure*)). (early adj4 (detect* or diagnos*)). predict*.
Embase	lung diseases, obstructive/ or exp. bronchitis/ or exp. pulmonary disease, chronic obstructive/ exp. telemonitoring/ or exp. telemedicine/ telerehabilitation/ exp. telephone telemetry/ or exp. telemetry/ exp. ambulatory monitoring/ computer program/ or exp. communication software/ or exp. mobile application/ exp. computer interface/ heart rate variability/ or exp. heart rate/ exp. pulse rate/ or exp. “heart rate and rhythm”/ exp. breathing/ or exp. breathing rate/ exp. oximetry/ or exp. measurement/ or exp. pulse oximetry/ exp. oxygen breathing/ exp. early diagnosis/	emphysema$. bronchiti$. (obstruct$ adj3 (pulmonary or lung$ or airway$ or airflow$ or bronch$ or respirat$)). (copd or coad or cobd or aecb). (telemonitor* or tele-health* or telehealth* or telemedicine or tele-medicine or tele-monitor*). (e-health or ehealth or m-health or mhealth or mobile health). (telemetr* or tele-metr*). ((respirat* or breath*) adj3 rate*). oximetr*. SPO_2_. Oxygen (physiological adj4 (variable* or measure*)). (early adj4 (detect* or diagnos*)). predict*. (monitoring adj4 (ambulatory or home$)). Domiciliary. (software* or app? or iphone or ipad or android or smartphone* or smart-phone*). (exacerbat* or deteriorat*). ((heart* or pulse* or cardiac) adj3 rate*).
AMED	pulmonary disease chronic obstructive/ or bronchitis/ or pulmonary emphysema/ or lung diseases obstructive/ telemedicine/ home care services/ internet/ or exp. computers/ or software/ disease progression/ heart rate/ Pulse/ exp. Respiration/ Oxygen/ monitoring physiologic/ or respiratory function tests/ diagnosis/	emphysema$. bronchiti$. (obstruct$ adj3 (pulmonary or lung$ or airway$ or airflow$ or bronch$ or respirat$)). (copd or coad or cobd or aecb). (telemonitor* or tele-monitor* or tele-health* or telehealth* or telemedicine or tele-medicine or tele-rehabilitat* or telerehabilitat*). (e-health or ehealth or m-health or mhealth or mobile health). (telemetr* or tele-metr*). ((monitoring adj4 (ambulatory or home$)). Domiciliary. (software* or app? or iphone or ipad or android or smartphone* or smart-phone*). (exacerbat* or deteriorat*). ((heart* or pulse* or cardiac) adj3 rate*). ((respirat* or breath*) adj3 rate*). oximetr*. SPO_2_. oxygen. (physiological adj4 (variable* or measure*)). (early adj4 (detect* or diagnos*)). predict*.
CINAHL	(MH “Lung Diseases, Obstructive”) OR (MH “Bronchitis+”) OR (MH “Emphysema”) OR (MH “Pulmonary Disease, Chronic Obstructive+”) (MH “Telenursing”) OR (MH “Telepathology”) OR (MH “Remote Consultation”) OR (MH “Telemedicine”) OR (MH “Telehealth”) (MH “Telemetry”) (MH “Ambulatory Care”) (MH “Software”) OR (MH “Communications Software+”) OR (MH “Mobile Applications”) OR (MH “User-Computer Interface+”) (MH “Pulse”) OR (MH “Heart Rate”) (MH “Wireless Communications”) OR (MH “Telephone+”) OR (MH “Instant Messaging”) (MH “Respiratory Rate”) OR (MH “Respiratory Sounds”) (MH “Respiration+”) (MH “Oximetry+”) (MH “Oximeters+”) (MH “Oxygen”) (MH “Oxygenation”) OR (MH “Oxygen Saturation”) (MH “Monitoring, Physiologic”) (MH “Early Diagnosis”)	TX emphysema* TX bronchiti* TX (copd or coad or cobd or aecb) TX (obstruct* n3 (pulmonary or lung* or airway* or airflow* or bronch* or respirat*)) TX (telemonitor* or tele-monitor* or tele-health* or telehealth* or telemedicine or tele-medicine or tele-rehabilitat* or telerehabilitat*) TX (e-health or ehealth or m-health or mhealth or “mobile health”) TX (telemetr* or tele-metr*) TX monitoring n4 (ambulatory or home*) TX Domiciliary TX (app# or iphone or ipad or android or smartphone* or smart-phone*) OR TI software* OR AB software* TX (exacerbat* or deteriorat*) TX ((heart* or pulse* or cardiac) n3 rate*) TX (respirat* or breath*) n3 rate* TX oximetr* TX SPO_2_ TX oxygen TX (physiological n4 (variable* or measure*)) TX (early n4 (detect* or diagnos*)) TX predict*
Cochran	[mh “lung diseases, obstructive”] [mh bronchitis] [mh “pulmonary disease, chronic obstructive”] [mh telemedicine] [mh telerehabilitation] [mh Telemetry] [mh “Monitoring, Ambulatory”] [mh software] [mh “mobile applications”] [mh “user-computer interface”] [mh “heart rate”] [mh pulse] [mh “respiratory rate”] [mh Respiration] [mh Oximetry] [mh Oxygen] [mh “early diagnosis”]	COPD emphysema* bronchiti* (obstruct* near/3 (pulmonary or lung* or airway* or airflow* or bronch* or respirat*)) (copd or coad or cobd or aecb) (telemonitor* or tele-monitor* or tele-health* or telehealth* or telemedicine or tele-medicine or tele-rehabilitat* or telerehabilitat*) (e-health or ehealth or m-health or mhealth or mobile health) (telemetr* or tele-metr*) (monitoring near/4 (ambulatory or home*)) Domiciliary (software* or app or apps or iphone or ipad or android or smartphone* or smart-phone*) (exacerbat* or deteriorat*) ((heart* or pulse* or cardiac) near/3 rate*) ((respirat* or breath*) near/3 rate*) oximetr* SPO_2_ Oxygen (physiological near/4 (variable* or measure*)) (early near/4 (detect* or diagnos*)) predict*

**Table A3:** Excluded Studies.

First Author	Study Title	Reason
Malliopoulos, C., 2008	Continuous mobile services for healthcare: The health wear project	Article not available and no response from the author
Antoniades, N.C., 2012	Pilot study of remote telemonitoring in COPD	No physiological data shown and it does not address the prediction of COPD exacerbation
Jensen, M.H., 2012	Clinical impact of home telemonitoring on patients with chronic obstructive pulmonary disease	Not relevant (evaluated the impact of tele-health on patients, not in predicting exacerbation)
Jakobsen, A.S., 2013	Hospital-admitted COPD patients treated at home using telemedicine technology in The Virtual Hospital Trial: methods of a randomized effectiveness trial	Recruited non-stable COPD patients for preventing readmission
Jehn, M., 2013	Tele-monitoring reduces exacerbation of COPD in the context of climate change-a randomized controlled trial	Looked at the association between the weather and exacerbation.
Pinnock, H., 2013	Effectiveness of telemonitoring integrated into existing clinical services on hospital admission for exacerbation of chronic obstructive pulmonary disease: researcher blind, multicentre, randomised controlled trial	No physiological variation reported and not for predicting exacerbation
San Miguel, K., 2013	Telehealth remote monitoring for community-dwelling older adults with chronic obstructive pulmonary disease	No physiological variation reported and not for predicting exacerbation
Schou, Lone, 2013	A randomised trial of telemedicine-based treatment versus conventional hospitalisation in patients with severe COPD and exacerbation—Effect on self-reported outcome	Not for predicting exacerbation and recruited non-stable COPD patients
van der Heijden, M., 2013	An autonomous mobile system for the management of COPD	Designing a mobile system
Zhang, J., 2013	MIOTIC study: A prospective, multicenter, randomized study to evaluate the long-term efficacy of mobile phone-based internet of things in the management of patients with stable COPD	No physiological variation reported and not for predicting exacerbation
Ding, H., 2014	A pilot study of a mobile-phone-based home monitoring system to assist in, remote interventions in cases of acute exacerbation of COPD	Did not report any monitored physiological data
Ko, F.W.S., 2014	COPD care programme can reduce readmissions and in-patient bed days	Recruited non-stable COPD patients
Minami S., 2014	Ambulatory pulse oximetry monitoring in Japanese COPD outpatients not receiving oxygen therapy	Monitored the patient’s SPO_2_% for a 24 h period only.
Jakobsen, A.S., 2015	Home-Based Telehealth Hospitalization for Exacerbation of Chronic Obstructive Pulmonary Disease: Findings from “The Virtual Hospital” Trial	Recruited non-stable COPD patients
Ringbaek, T., 2015	Effect of telehealthcare on exacerbations and hospital admissions in COPD: A randomised controlled trial	No physiological variation reported and not for predicting exacerbation
